# Remarks on Congestive Fever

**Published:** 1849

**Authors:** C. A. Hathwell

**Affiliations:** Virginia, Cass county, Illinois


### ARTICLE VI.

*Remarks on Congestive Fever.* By C. A. Hathwell, M. D., of Virginia, Cass county, Illinois.

Congestive Fever is one of the most formidable diseases that medical men have to encounter, and imperatively demands the soundest judgment, watchfulness, promptness, and energy on the part of the attending physician. It is not my intention to furnish a treatise on this disease, but to state in as concise a manner as possible, the result of treatment in some cases that came under my care.

I shall confine myself to such articles as I have found most serviceable.

According to circumstances I have generally commenced with an active cathartic. Purgatives clear the alimentary canal of all morbid accumulations and relieve congestion. To obtain their full effect, I administer them daily. I usually employ calomel and jalap, with a small portion of tartarized antimony, worked off with oil or the neutral salts.

The bowels are generally loaded. The patient has strong sensations of internal heat, and there is also considerable gastric distress. These circumstances seem to imperately to call for such evacuations, and every practitioner knows how much relief and comfort is afforded by them. No one conversant with the modus operandi of purges will fear their producing debility. At this period they not only relieve the stomach, but also congestion of the liver, head, and other important organs, upon the principle of revulsion. Nothing is better established than that, when the alimentary canal is oppressed with accumulations of feculent matter, the evacuation of this matter relieves the irritation of the system and adds new vigor to the body. As one of the auxiliaries, I place great confidence in cold applications; sponging the surface is a favorite remedy. I employ water, vinegar and water, brandy, &c. I do not believe they act by merely lessening the heat of the body, that they operate to a certain extent in this way I think certain, but they are more beneficial from the positive healthy action they impart to the system. As soon as practicable, I place my patient under the influence of large and repeated doses of quinine—it is, in fact, the only remedy we can rely on with confidence. But in certain cases during the cold stage, when I have found all the external applications, together with the most powerful and diffusible stimulants used internally, fail to bring about a reaction, I have found the use of ice to produce the most salutary effects. When I have met my patient in a state of great jactitation, complaining of intense internal heat, insatiable thirst, oppression in respiration, cold surface, pulseless, and with all the symptoms characteristic of this disease, I have found ice all powerful in producing reaction and restoring an equilibrium. My plan has been to break the ice into small pieces and set it by the bed side of my patient, force him to swallow it as fast as possible until the stomach is literally filled with solid pieces of it. The revulsive influence of ice has, in several instances, perfectly surprised me; but when we reflect upon the pathology of this disease, the congestion of the stomach and the great central accumulation and engorgement of the heart, liver, and large veins, the modus operandi may be easily made out. As soon as reaction is established, I look upon the case as completely under the control and management of quinine. In protracted cases of collapse, when the blood has become vitiated for the want of oxygenation, perhaps there is but little reliance to be placed in any remedy.

